# Potential of high dimensional radiomic features to assess blood components in intraaortic vessels in non-contrast CT scans

**DOI:** 10.1186/s12880-021-00654-9

**Published:** 2021-08-12

**Authors:** Scherwin Mahmoudi, Simon S. Martin, Jörg Ackermann, Yauheniya Zhdanovich, Ina Koch, Thomas J. Vogl, Moritz H. Albrecht, Lukas Lenga, Simon Bernatz

**Affiliations:** 1 Department of Diagnostic and Interventional Radiology, University Hospital Frankfurt, Theodor-Stern-Kai 7, 60590 Frankfurt am Main, Germany; 2 Department of Molecular Bioinformatics, Institute of Computer Science, Johann Wolfgang Goethe-University, Robert-Mayer-Str. 11-15, 60325 Frankfurt am Main, Germany

**Keywords:** Radiomics, Blood, Anemia, Artificial intelligence, CT

## Abstract

**Background:**

To assess the potential of radiomic features to quantify components of blood in intraaortic vessels to non-invasively predict moderate-to-severe anemia in non-contrast enhanced CT scans.

**Methods:**

One hundred patients (median age, 69 years; range, 19–94 years) who received CT scans of the thoracolumbar spine and blood-testing for hemoglobin and hematocrit levels ± 24 h between 08/2018 and 11/2019 were retrospectively included. Intraaortic blood was segmented using a spherical volume of interest of 1 cm diameter with consecutive radiomic analysis applying PyRadiomics software. Feature selection was performed applying analysis of correlation and collinearity. The final feature set was obtained to differentiate moderate-to-severe anemia. Random forest machine learning was applied and predictive performance was assessed. A decision-tree was obtained to propose a cut-off value of CT Hounsfield units (HU).

**Results:**

High correlation with hemoglobin and hematocrit levels was shown for first-order radiomic features (*p* < 0.001 to *p* = 0.032). The top 3 features showed high correlation to hemoglobin values (*p*) and minimal collinearity (r) to the top ranked feature Median (*p* < 0.001), Energy (*p* = 0.002, r = 0.387), Minimum (*p* = 0.032, r = 0.437). Median (*p* < 0.001) and Minimum (*p* = 0.003) differed in moderate-to-severe anemia compared to non-anemic state. Median yielded superiority to the combination of Median and Minimum (*p*(AUC) = 0.015, *p*(precision) = 0.017, *p*(accuracy) = 0.612) in the predictive performance employing random forest analysis. A Median HU value ≤ 36.5 indicated moderate-to-severe anemia (accuracy = 0.90, precision = 0.80).

**Conclusions:**

First-order radiomic features correlate with hemoglobin levels and may be feasible for the prediction of moderate-to-severe anemia. High dimensional radiomic features did not aid augmenting the data in our exemplary use case of intraluminal blood component assessment.

*Trial registration* Retrospectively registered.

**Supplementary Information:**

The online version contains supplementary material available at 10.1186/s12880-021-00654-9.

## Background

Radiomics is a term coined for computational quantitative imaging analysis and has been shown to aid in clinical decision making [1]. Radiomics extracts a large number of quantitative data from medical images that can provide surrogate information on biochemical and pathophysiological processes [2, 3]. The technique has been successfully applied to evaluate tumor characteristics non-invasively [4]. While several studies showed the benefits of radiomics in solid tissue and predominantly cancer research [5–7], its potential to assess flowing structures and moving tissues using static acquisition protocols has not yet been investigated.

Acute and chronic blood loss might not only be surrogates of yet undiagnosed diseased which require further workup but also might be considered as an illness itself which requires hemostasis management [8, 9]. In emergency patients with acute blood loss, fast assessment of a multitude of blood components, for example hemoglobin and hematocrit levels is essential [10, 11]. In 2002, the World Health Organization has attributed anemia as one of the most relevant risk factors leading to high mortality and morbidity [12, 13]. During hospitalization, phlebotomy is the current standard of screening for a load of blood components [14]. Blood samples are usually easily obtained, but the procedure can be time consuming in some cases [15]. Non-invasive screening of blood components in a clinically indicated CT may yield the potential to assess specific blood components in order to focus invasive testing on pre-filtered components and patients to reduce workload and costs of laboratory analyses [16].

Computed tomography (CT) is a commonly used imaging modality in hospitalized patients and provides non-invasive assessment of tissue morphology. Previous studies have suggested that simple attenuation measurements in CT scans correlate with hemoglobin and hematocrit levels and may be useful in predicting anemia [17–19]. Foster et al. analyzed regions of interest of the left ventricular cavity, aorta and interventricular septum and concluded that the visualization of the interventricular septum in unenhanced CT scans suggests anemia [18]. In their study from 2001, Collins et al. examined regions of interest placed within the lumen of the aorta and inferior vena cava. The authors found a significant correlation between patients’ hemoglobin measurements and aortic ROI values, in particular simple attenuation measurements [19].

By extracting a variety of mineable image features, radiomics can provide additional, higher dimensional data that can be employed to improve decision support. Current radiomic research promotes the impression that radiomic features are potentially applicable to augment data in a variety of diseases [1]. However, the potential of radiomic features to assess the intraluminal blood compartment to predict specific components has not yet been sufficiently evaluated. Since prior studies have investigated the validity of simple attenuation measurements in CT scans for the prediction of blood levels, the aim of this study was to assess the predictability of hemoglobin and hematocrit levels using high dimensional radiomic features in non-contrast enhanced CT scans.

## Methods

### Patient selection

The local Ethics committee approved this retrospective study (project number: 20–689, Goethe University Frankfurt am Main, Germany) and waived informed written consent.

**Fig. 1:**
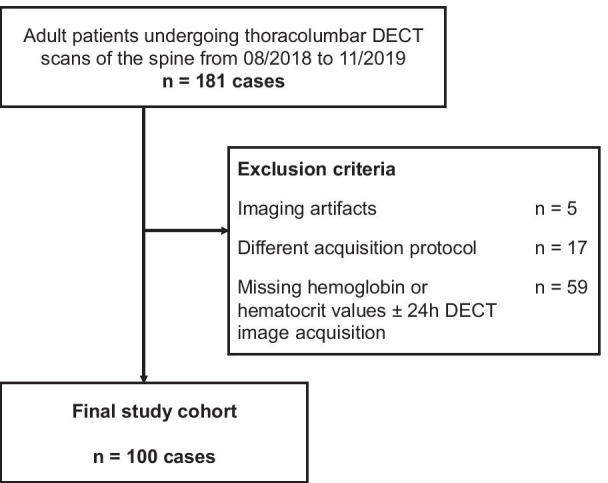
CLAIM flowchart of patient inclusion into the study. CLAIM, Checklist for Artificial Intelligence in Medical Imaging; DECT, dual-energy computed tomography

A total of 181 consecutive patients (female, 54; male, 46; age, 69 (19–94) years) who underwent non-contrast dual-energy CT imaging of the thoracolumbar spine between 08/2018 and 11/2019 were screened for study inclusion. Inclusion criteria were (I) > 18 years of age, (II) thoracolumbar region, (III) 1 mm 90 kV series, (IV) hemoglobin and hematocrit values ± 24 h CT examination. Exclusion criteria were (I) different acquisition protocol, (II) signs of active bleeding, (III) imaging artifacts. All clinical data were obtained in clinical routine. 100 patients met the criteria and were evaluated. Figure 1 shows the flowchart of patient inclusion. Table 1 depicts patient characteristics.

**Table 1:** Patients characteristics and baseline demographics

Parameters	Value
Patients	100
Female	54
Male	46
Median age (y)*	69 (19–94)
Maximum time (h) between blood test / CT scan	± 24
Mean hemoglobin [mg/dL]**	11.82 (2.39, 12.29, 11.35)
Mean hematocrit [%]**	34.80 (6.72, 36.14, 33.47)

If not state otherwise, the numbers depict absolute numbers

CT, computed tomography; h, hours; y, years

^*^Data in round parenthesis are min/max values (interquartile range)

^**^Data in round parenthesis are standard deviation and ± 95% confidence interval

### CT acquisition protocol

Examinations were performed using a third-generation, dual-source, dual-energy CT system (Somatom Force; Siemens Healthineers, Forchheim, Germany). The non-contrast acquisition protocol operated the X-ray tubes at different kilovoltage settings (tube A, 90kVp, 260.9 ± 88mAs, reference 300mAs; tube B, Sn150kVp [0.64 mm tin filter], 157.8 ± 41.9mAs, reference 188mAs). The dual-energy protocol (rotation time, 0.5 s; pitch, 0.6; collimation, 2 × 192 × 0.6) included automatic attenuation-based tube current modulation (CARE Dose 4D; Siemens Healthineers) with a mean volume CT dose index of 12.2 ± 3.8 mGy and a mean dose-length product of 507.4 ± 255.8 mGy × cm.

### Image reconstruction

We applied the 90kVp images as they are reconstructed using isotropic voxels in clinical routine (axial, section thickness 1 mm and increment of 1 mm) with a dedicated dual-energy medium-soft convolution kernel (Qr40, advanced model-based iterative reconstruction [ADMIRE] level of 3). For the consecutive quantitative analysis, the image stack was extracted in Digital Imaging and Communications in Medicine (DICOM) format.

### Radiomic analysis

**Fig. 2:**
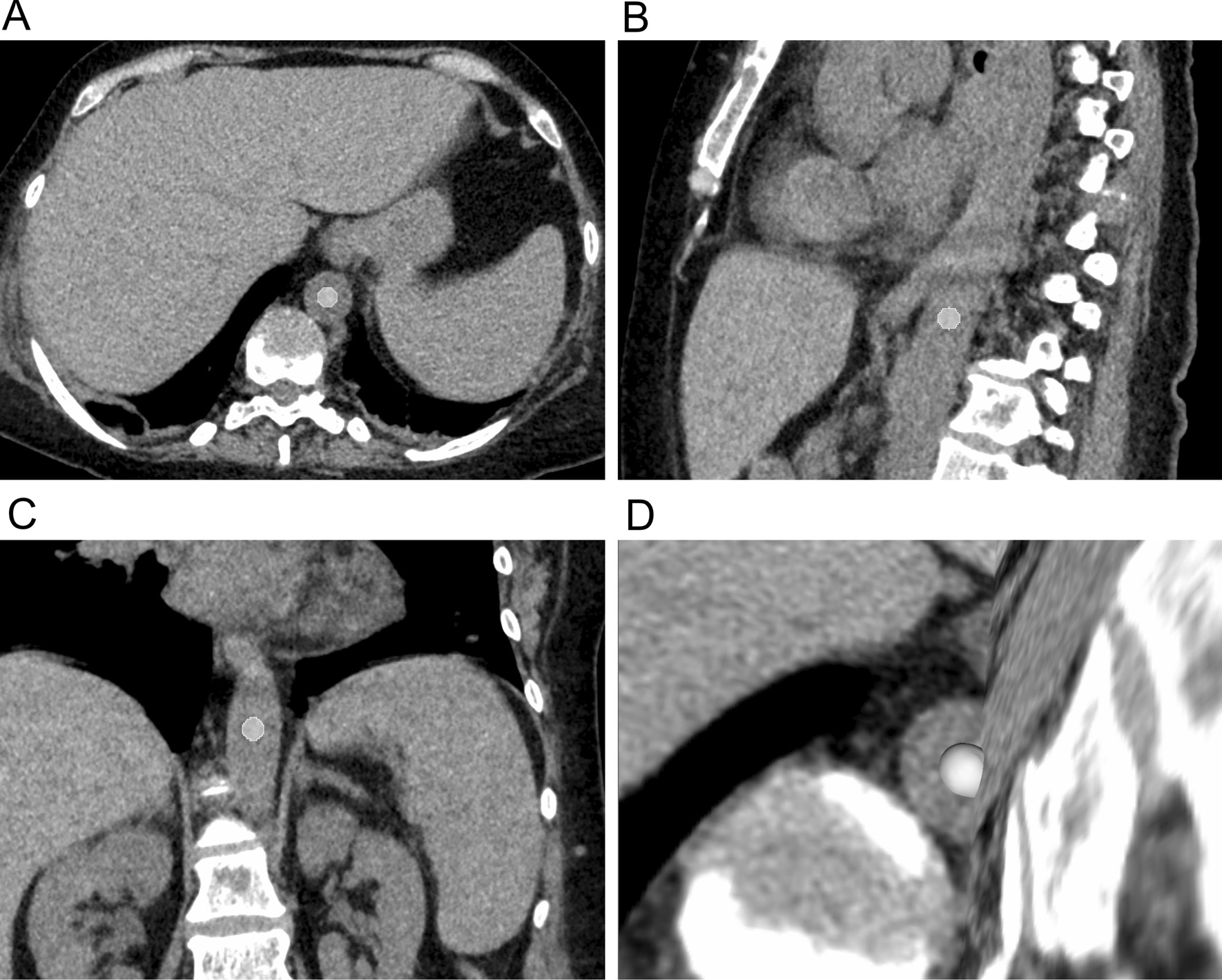
Representative images of the measurement technique. Axial (**a**), sagittal (**b**) and coronal (**c**) plane with 3D-volume rendering (**d**) of a standard volume of interest (VOI) placement is shown in a patient with a hemoglobin and hematocrit level of 7.2 g/dL and 22.4%, respectively. A spherical VOI with 1 cm in diameter was placed within the lumen of the thoracoabdominal aorta as described in detail in the materials and methods section

The 3D Slicer software platform (http://slicer.org, version 4.9.0) was applied to visualize and process the DICOM image stack [2, 20]. For segmentation, a radiologist (SM) with two years of experience manually defined a spheric volume of interest (VOI, 1.0 cm diameter) centrically in the aorta of the thoracolumbar region, sparing the aortic wall and visual artifacts (Fig. 2). Based on the findings of Collins et al. [19], we chose a small section of the intraaortic lumen for measurement to imitate a virtual blood sample*.* All VOIs were reviewed by a second radiologist (SB, two years of experience). Both radiologists were blinded to the laboratory results. Prior to feature extraction we did not perform further image manipulation as the Imaging Biomarker Standardization (IBSI) does currently not cover image preprocessing and we did perform our analysis on isotropic 1 mm × 1 mm voxels [21]. The open-source package PyRadiomics was used as extension within 3D Slicer to extract the radiomic features [2, 20, 22]. We extracted all standard features from seven feature classes: First Order Statistics, Shape-based, Gray Level Co-occurrence Matrix (GLCM), Gray Level Run Length Matrix (GLRLM), Gray Level Size Zone Matrix (GLSZM), Gray Level Dependence Matrix (GLDM), Neighbouring Gray Tone Difference Matrix (NGTDM), obtaining 105 features / VOI (http://pyradiomics.readthedocs.io) [22]. PyRadiomics was operated using the default settings (bin width 25, enforced symmetrical GLCM, http://pyradiomics.readthedocs.io) [22, 23]. As we used a spherical 1 cm VOI for segmentation, shape features were excluded for analysis, obtaining 93 features, further referred to as “all features” for statistical analysis. To assess the methodological quality of our study, we used the Radiomic Quality Score (Additional file 1).

### Statistical analysis and machine learning

We performed radiomic feature reduction and selection to match hemoglobin [g/dL] and hematocrit [%] values. Correlation analysis of all features was performed against hemoglobin and hematocrit values [24]. We ranked the features according to the obtained *p*-value of the correlation analysis. The lower the *p*-value, the higher the ranking. Next, we used inter-correlation analysis of the features which showed significant correlation for both hemoglobin and hematocrit levels to test for collinearity [1]. Features with a collinearity of r < 0.5 were selected for further analysis. Next, we analyzed the obtained radiomic features set to differentiate moderate-to-severe anemic state. Moderate-to-severe anemia is defined by a cut-off value of hemoglobin ≤ 10–11 g/dL depending on age and gender [25–28]. For our primarily methodologically driven study we aimed to choose a uniform definition of moderate-to-severe anemia and therefore defined a cut-off value of hemoglobin ≤ 10 g/dL for our cohort as previously proposed. We built two machine learning models based on random forest (RF) algorithms to predict moderate-to-severe anemia. The predictive power was assessed by receiver operating characteristics (ROC) curves with Monte Carlo cross-validation with 100 random splits. Each run randomly drew 70% of the samples for training and tested the model with the remaining independent 30% of the data. We obtained the area under the curve (AUC), precision and accuracy. To analyze the variation of predictive power we applied a two-tailed student’s t test of the cross-validated measurements. Machine learning algorithms and visualization of the decision tree were conducted in Python 3.7 using the open-source scikit-learn 0.21.3 packages RandomForestClassifier (n_estimators = 100, max_depth = 1/(2) for one/(two) feature(s)) for RF analysis with prior normalization of features employing StandardScaler (https://scikit-learn.org/) and DecisionTreeClassifier with criterion = gini and max_depth equivalent to the RF-analysis [29]. Further statistical analyses were performed using Prism 6.0 (GraphPad software) and JMP 14 (SAS, Cary, U.S.A.). The significance values were indicated as followed: *p < 0.05; **p < 0.01; ***p < 0.001. The respective table and figure legends give detailed information about the statistical tests.

## Results

From all radiomic features, 9 features revealed significant correlation (*p* < 0.001–*p* = 0.032) to hemoglobin and hematocrit levels with Median (*p* < 0.001) as the highest ranked feature (Table 2). The features were found to be part of one feature class, the first-order statistics (Table 2). Grey Level Non Uniformity, a feature of the GLSZM feature class, showed correlation to hematocrit levels, but no significance to hemoglobin levels (Table 2). It was therefore excluded for further analysis.

**Table 2:** Top 20 radiomic features with highest variable importance based on measurement of correlations with hemoglobin and hematocrit values

Features	Hemoglobin *p*-value	Hematocrit *p*-value
firstorder-Median	** < 0.001**	** < 0.001**
firstorder-Mean	** < 0.001**	** < 0.001**
firstorder-RootMeanSquared	** < 0.001**	** < 0.001**
firstorder-TotalEnergy	** < 0.001**	** < 0.001**
firstorder-90Percentile	** < 0.001**	** < 0.001**
firstorder-10Percentile	** < 0.001**	** < 0.001**
firstorder-Maximum	** < 0.001**	** < 0.001**
firstorder-Energy	**0.002**	**0.001**
firstorder-Minimum	**0.032**	**0.014**
glszm-GrayLevelNonUniformity	0.052	**0.023**
glszm-LowGrayLevelZoneEmphasis	0.069	0.074
glcm-MaximumProbability	0.083	0.108
glrlm-ShortRunLowGrayLevelEmphasis	0.101	0.109
glszm-SmallAreaLowGrayLevelEmphasis	0.083	0.115
glcm-Idmn	0.118	0.128
ngtdm-Contrast	0.149	0.135
glszm-SmallAreaEmphasis	0.094	0.138
glrlm-LowGrayLevelRunEmphasis	0.124	0.141
glszm-SmallAreaHighGrayLevelEmphasis	0.162	0.149
gldm-LowGrayLevelEmphasis	0.133	0.15

Measurement of correlation of all radiomic features with hemoglobin and hematocrit levels obtained ± 24 h to the acquisition of the computed tomography images. Measurement of probability used for hypothesis testing is depicted as *p*-value. Significant values are labeled in bold font. Top 20 features are shown, sorted according to the hematocrit p-value and with the matching hemoglobin *p*-value

**Fig. 3:**
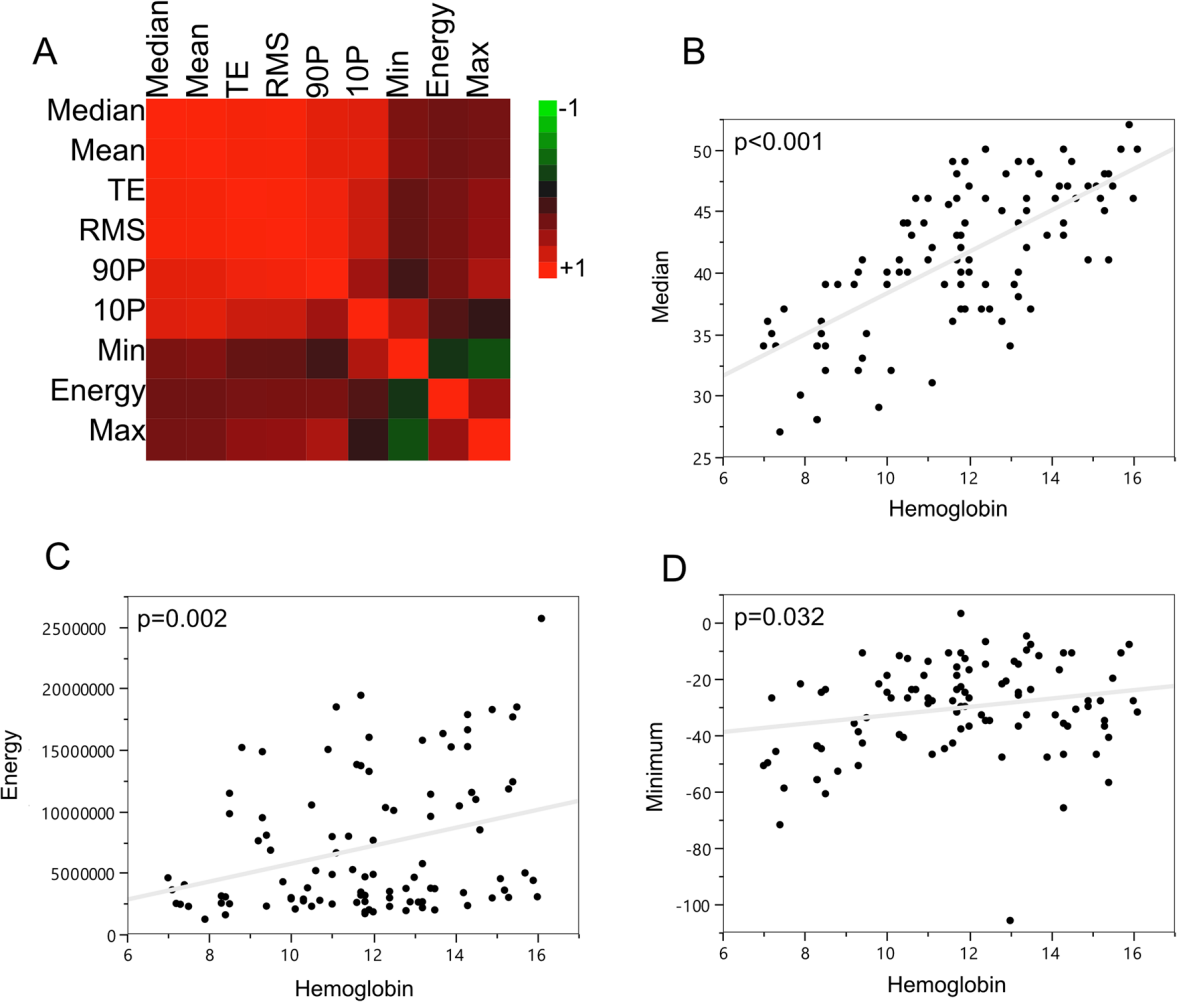
Analysis of radiomic features that are significantly correlated with hemoglobin and hematocrit levels. The matrix of correlations of the selected radiomic features with highest correlation to the hemoglobin [g/dL] and hematocrit [%] levels obtained ± 24 h to computed tomography images are shown (**a**). Exemplary scatter plots of the correlation of hemoglobin values with the prioritized top 3 radiomic features are shown (**b**–**d**). All depicted features belong to the feature class of first-order statistics. 10P = 10 Percentile; 90P = 90 Percentile; Max = Maximum; Min = Minimum; RMS = Root Mean Squared; TE = Total Energy

The selected features showed a high degree of collinearity (Fig. 3a, Table 3). Energy (r = 0.387), Maximum (r = 0.411) and Minimum (r = 0.437) were found to be the least correlated features to Median (Table 3). As Maximum revealed collinearity with Energy (r = 0.568) it was excluded for further analysis. We therefore obtained the top 3 features to correlate with hemoglobin and hematocrit levels: Median (*p* < 0.001, Fig. 3b), Energy (*p* = 0.002, Fig. 3c) and Minimum (*p* = 0.032, Fig. 3d).

**Table 3:** Matrix of correlations of radiomic features with significant correlation with hemoglobin and hematocrit levels

	Firstorder-Median	firstorder-Energy	firstorder-TotalEnergy	firstorder-Maximum	firstorder-RootMeanSquared	firstorder-90Percentile	firstorder-Minimum	firstorder-10Percentile	firstorder-Mean
firstorder-Median	1.000	0.387	0.971	0.411	0.977	0.891	0.437	0.869	0.993
firstorder-Energy	0.387	1.000	0.422	0.568	0.427	0.431	− 0.139	0.253	0.388
firstorder-TotalEnergy	0.971	0.422	1.000	0.525	0.992	0.947	0.339	0.783	0.973
firstorder-Maximum	0.411	0.568	0.525	1.000	0.541	0.646	− 0.273	0.116	0.422
firstorder-RootMeanSquared	0.977	0.427	0.992	0.541	1.000	0.961	0.334	0.781	0.980
firstorder-90Percentile	0.891	0.431	0.947	0.646	0.961	1.000	0.186	0.598	0.894
firstorder-Minimum	0.437	− 0.139	0.339	− 0.273	− 0.334	0.186	1.000	0.665	0.468
firstorder-10Percentile	0.869	0.253	0.783	0.116	0.781	0.598	0.665	1.000	0.887
firstorder-Mean	0.993	0.388	0.973	0.422	0.980	0.894	0.468	0.887	1.000

Multivariate measurements of correlations of radiomic features that are significantly correlated with hemoglobin and hematocrit levels

**Fig. 4:**
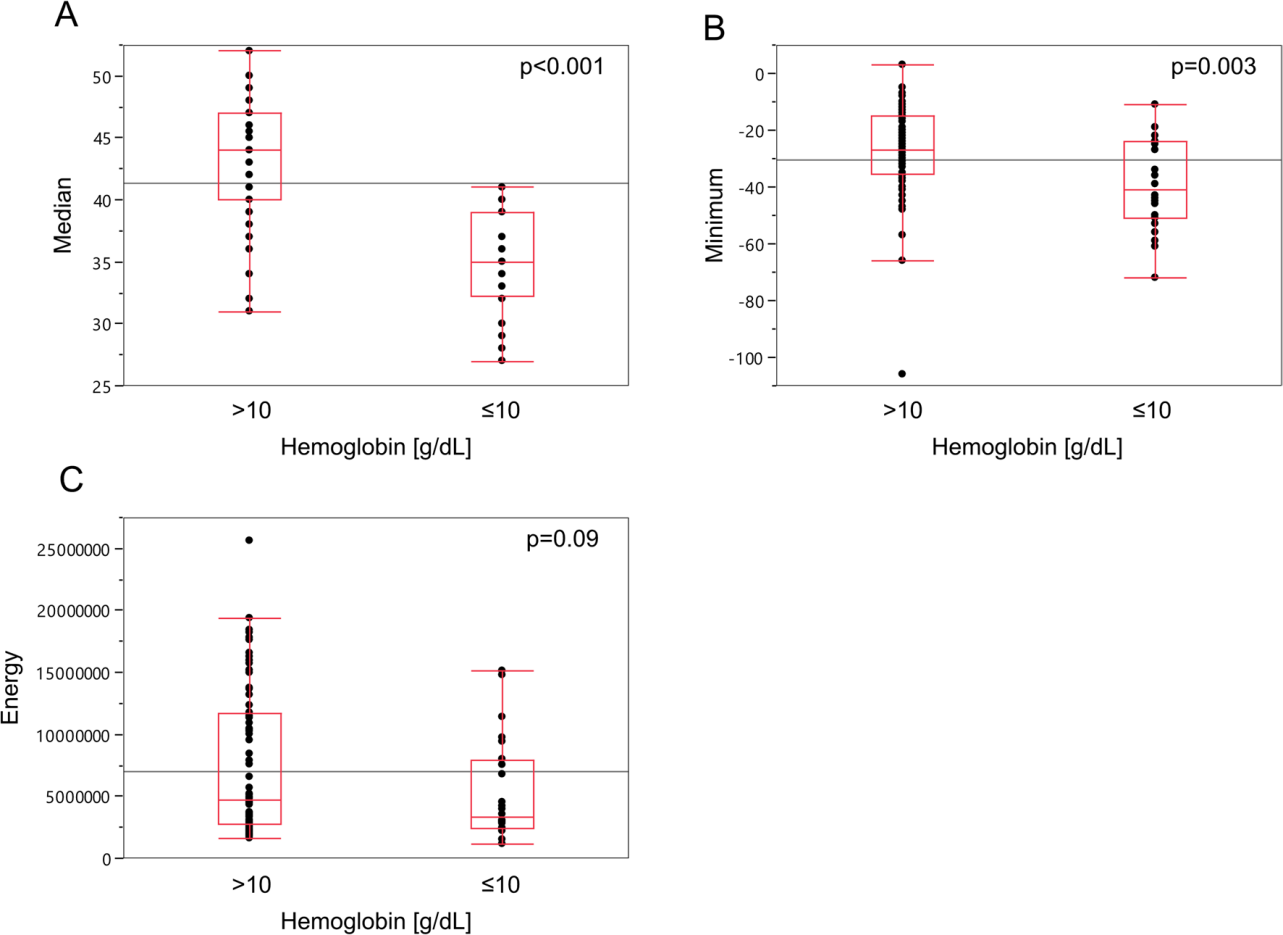
Radiomic features to decipher moderate-to-severe anemia. Box-Whisker Plots for the radiomic features median (**a**), minimum (**b**) and energy (**c**) versus hemoglobin levels are shown. Hemoglobin values were split according to the threshold of 10 g/dL to differentiate moderate-to-severe anemia [25–28]. Statistical analyses are depicted using two-tailed student’s *t*-test

Radiomic analysis of intraaortic blood to differentiate a threshold of hemoglobin level of 10 mg/dL revealed significant difference in the radiomic features Median (*p* < 0.001, Fig. 4a) and Minimum (*p* = 0.003, Fig. 4b) whereas Energy did not reach the level of significance (*p* = 0.09, Fig. 4c) and was therefore excluded for the consecutive machine learning model development.

**Fig. 5:**
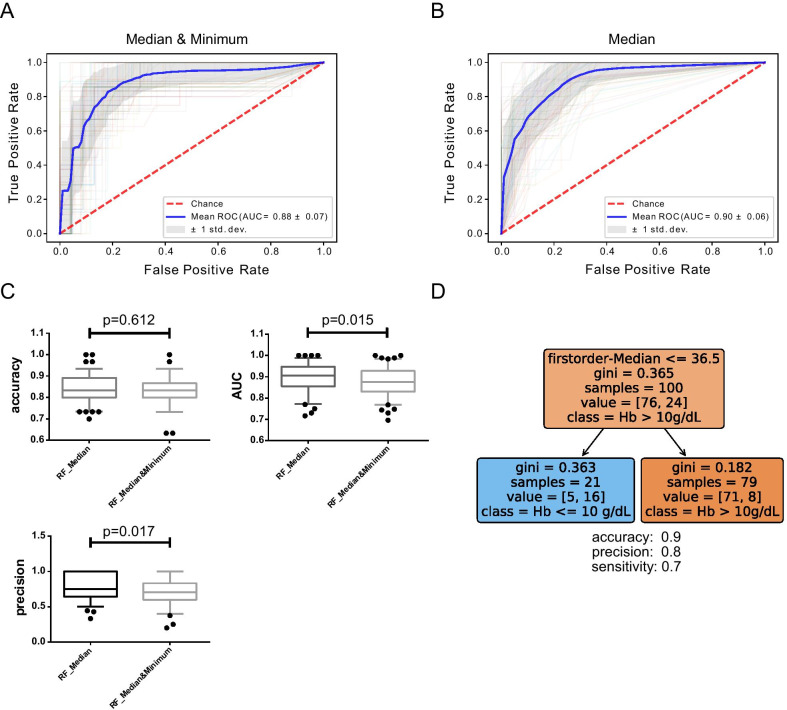
Median density measurement of Hounsfield units reveals the best working model to predict moderate-to-severe anemia. Analysis of prediction performance for moderate-to-severe anemia with 2 variant feature subsets applying random forest (RF) machine learning algorithms (**a**–**c**). Monte Carlo cross-validation with 100 random splits (colored lines represent each single measurement) receiver operating characteristics (ROC) curve analysis of the validation cohort with mean ROC curve (blue) and ± 1 standard deviation (grey area) are shown for Median and Minimum (**a**) or Median only (**b**). RF maximum depth was 2 (**a**) and 1 (**b**). **c** The Box-Whisker Plots with 5–95% percentile for both cross-validated prediction models with the respective accuracy, area under the curve (AUC) and precision. Two-tailed, unpaired student’s *t*-test was applied for model comparison (**c**, *p*-values). **d** A decision tree with a depth of 1 for firstorder-Median. The gini value measures the impurity of the group. The decision tree minimizes the measure of impurity by a bisection of the group of 100 patients into two groups, one with 21 patients and a second with 79. The so-called gini gain, i.e., the sum of gini values of the child nodes weighted by the number of their members, becomes optimal for a selection threshold 36.5 of the firstorder-Median

A random forest based, Monte Carlo cross-validated machine learning approach with 100 random splits was conducted applying either Median and Minimum features (Fig. 5a, AUC 0.88 ± 0.07) or Median feature only (Fig. 5b, AUC 0.90 ± 0.06) for model building. Application of the single radiomic feature Median was superior to its combination with the feature Minimum with regard to AUC and precision measurements whereas no difference was found with regard to model accuracy (Fig. 5c, accuracy *p* = 0.612, AUC *p* = 0.015, precision *p* = 0.017).

We obtained a decision tree based on the radiomic feature Median (Fig. 5d). With a cutoff value of ≤ 36.5 Hounsfield Units (HU) in an independent train/test set of patients drawn at random, we obtained a test accuracy of 0.90, precision of 0.80 and sensitivity of 0.7 to predict moderate-to-severe anemic state.

## Discussion

In this study, we examined the potential of high dimensional radiomic features to assess components of the moving blood compartment. We assumed that hemoglobin and hematocrit may be the most promising and easily non-invasively accessible values and may aid predicting moderate-to-severe anemia. Examining 100 non-enhanced CT scans, we demonstrated correlation of first-order radiomic features with hemoglobin and hematocrit levels. We could obtain a cut-off value of ≤ 36.5 HU for Median to predict moderate-to-severe anemia with an accuracy of 0.90 and a precision of 0.80. We could show that higher dimensional radiomic features did not augment simple first order radiomic features. Based on our findings, we conclude that besides its benefit to evaluate solid tissue and tumor characteristics non-invasively, the application of higher dimensional radiomic features to analyze flowing structures such as the blood system applying static CT images does not seem to be promising.

Our results regarding first order radiomic features are in accordance with previous studies investigating the potential of qualitative and quantitative measurements of CT density to differentiate between anemic and non-anemic conditions [17–19, 27, 30, 31]. In a study from 2003, Foster et al. concluded that visualization of the interventricular septum in unenhanced CT scans should suggest anemia [18]. In contrast to this subjective approach suggested by Foster et al., Collins et al. described a quantitative approach by demonstrating a significant correlation between patients’ hemoglobin measurements and aortic ROI values [19].

In a study of 102 patients undergoing thoracic CT scans, the authors obtained mean attenuation measurements in the left ventricle which performed better than subjective reviewer analysis [27]. Another study revealed a correlation between mean attenuation values of the thoracic aorta and hemoglobin values [30]. Nevertheless, these studies did not include higher dimensional radiomic features, limiting their quantitative assessment to the mean value only [30].

Quantitative imaging data have been increasingly applied in the last years. Especially in cancer research, radiomics is a rapidly evolving research field [32, 33]. In contrast to results obtained from research of specific tissues or tumor types, our data suggest that the application of high dimensional radiomic features may not yield diagnostic value assessing flowing structures, such as specific components of the intraaortic blood stream in static clinical routine CT images. In our study, high dimensional radiomic features were inferior to simple first order statistic values to estimate hemoglobin or hematocrit values and they were not applicable to predict moderate-to-severe anemia. However, first-order histogram features did significantly correlate with hemoglobin and hematocrit values with promising predictive power of therapeutically relevant anemic state. Our results seem logical and reasonable as the “texture” of intravascular blood is not static but varies continuously with its flow, whereas first-order values should not be affected by the variation of distribution of individual intraaortic voxels.

Potential problems at each step of the radiomics workflow including image acquisition, image reconstruction, segmentation and pre-processing have already been described in literature [34]. In their study from 2020, Fornacon-Wood et al. argued that different acquisition protocols [35], image reconstruction algorithms, reconstruction parameters (kernel) [36] and number of grey levels used to discretize histogram [37] affect feature values and feature reproducibility. Our study suggests that these issues seem to be more relevant in moving and dynamic compartments as high dimensional radiomic features had no diagnostic power for the prediction of hemoglobin and hematocrit levels. This raises the question whether most of the measured texture in a non-contrast-enhanced CT blood pool may be the effect of imaging artifacts due to the laminar flow of the blood system rather than true data of biological components.

Our study has limitations that warrant discussion. Analyzing retrospective data with continuous patient enrollment, we cannot rule out a selection bias. We had a moderate bias towards females and the older population and cannot rule out that a more balanced study population might have altered the results. Depending on age and gender, moderate-to-severe anemia is defined by a cut-off value of hemoglobin ≤ 10–11 g/dL [25–28]. As previously described, we chose a uniform cut-off value of hemoglobin ≤ 10 g/dL for our primarily methodologically driven study but we cannot rule out that age, gender or pregnancy adjusted values might have altered the results. Our study design was limited to 100 patients and a bigger cohort might have been favorable. This bias might reduce generalizability of the results and the finally obtained cut-off value of 36.5 HU to differentiate moderate-to-severe anemic state. We restricted the patient inclusion to one dual-energy CT scanner to exclude inter-scanner variability and to include only reconstructions with 1 mm isotropic voxels, nevertheless, intra-scanner variability may have occurred. We used iterative reconstruction methods and cannot rule out an effect on radiomic metrics. We limited the region of VOI definition to the thoracolumbar region to have an adequate diameter of the aorta for VOI placement and to limit pulsation artifacts that might be present at the ascending thoracic aorta.

## Conclusions

CT is a commonly applied imaging modality for a multitude of diagnostic purposes and attenuation measurements of various degrees of complexity are easily performed. We obtained simple histogram and high dimensional radiomic features and could demonstrate that histogram radiomic features enable an accurate differentiation of moderate-to-severe anaemic state and non-anemic state employing non-enhanced CT scans. We emphasize that our results are the first to show that high dimensional radiomic features are inferior to simple histogram features and do not yield additional information for the assessment of components of intraluminal blood in our use case to assess hemoglobin and hematocrit levels. Based on our findings, we conclude that higher dimensional radiomic features do not seem to be useful to predict components of flowing structures using static CT images, probably as high dimensional radiomic features are based on texture analyses and “texture” of intravascular blood varies continuously with its flow. The application of radiomics may be limited to the assessment of solid tissues and tumor characteristics.

## Supplementary Information

**Additional file 1**. Calculation of the radiomics quality score for this study (https://radiomics.world/rqs) [38].

## Data Availability

The datasets used and/or analysed during the current study are available from the corresponding author on reasonable request.

## Abbreviations

AUC: Area under the curve
CT: Computed tomography
GLCM: Gray Level Co-occurrence Matrix
GLDM: Gray Level Dependence Matrix
GLRLM: Gray Level Run Length Matrix
GLSZM: Gray Level Size Zone Matrix
HU: Hounsfield Units
NGTDM: Neighboring Gray Tone Difference Matrix
ROC: Receiver operating characteristics
VOI: Volume of interest
